# Exploration of tissue fixation methods suitable for digital pathological studies of the testis

**DOI:** 10.1186/s40001-024-01921-5

**Published:** 2024-06-10

**Authors:** Pengxiang Tian, Zhan Yang, Changbao Qu, Xin Qi, Linlin Zhu, Guimin Hao, Yong Zhang

**Affiliations:** 1 Department of Urology, The Second Hospital of Hebei Medical University, 215 Heping W Rd, Shijiazhuang, 050000 China; 2 Department of Urology, National Cancer Center/National Clinical Research Center for Cancer/Cancer Hospital, Chinese Academy of Medical Sciences and Peking Union Medical College, Beijing, 100021 China; 3 Department of Urology, National Cancer Center/National Clinical Research Center for Cancer/Hebei Cancer Hospital, Chinese Academy of Medical Sciences, Langfang, 065001 Hebei China; 4 Department of Forensic Medicine, Hebei Key Laboratory of Forensic Medicine, Collaborative Innovation Center of Forensic Medical Molecular Identification, Hebei Medical University, Shijiazhuang, 050017 China; 5 Department of Reproductive Medicine, The Second Hospital of Hebei Medical University, 215 Heping W Rd, Shijiazhuang, 050000 China

**Keywords:** Mouse testis, Fixative, HE staining, IHC staining, PAS staining, Digital pathology

## Abstract

**Background:**

The way of testicular tissue fixation directly affects the correlation and structural integrity between connective tissue and seminiferous tubules, which is essential for the study of male reproductive development. This study aimed to find the optimal fixative and fixation time to produce high-quality testicular histopathological sections, and provided a suitable foundation for in-depth study of male reproductive development with digital pathology technology.

**Methods:**

Testes were removed from both sides of 25 male C57BL/6 mice. Samples were fixed in three different fixatives, 10% neutral buffered formalin (10% NBF), modified Davidson’s fluid (mDF), and Bouin’s Fluid (BF), for 8, 12, and 24 h, respectively. Hematoxylin and eosin (H&E) staining, periodic acid Schiff-hematoxylin (PAS-h) staining, and immunohistochemistry (IHC) were used to evaluate the testicle morphology, staging of mouse seminiferous tubules, and protein preservation. Aperio ScanScope CS2 panoramic scanning was used to perform quantitative analyses.

**Results:**

H&E staining showed 10% NBF resulted in an approximately 15–17% reduction in the thickness of seminiferous epithelium. BF and mDF provided excellent results when staining acrosomes with PAS-h. IHC staining of synaptonemal complexes 3 (Sycp3) was superior in mDF compared to BF-fixed samples. Fixation in mDF and BF improved testis tissue morphology compared to 10% NBF.

**Conclusions:**

Quantitative analysis showed that BF exhibited a very low IHC staining efficiency and revealed that mouse testes fixed for 12 h with mDF, exhibited morphological details, excellent efficiency of PAS-h staining for seminiferous tubule staging, and IHC results. In addition, the morphological damage of testis was prolonged with the duration of fixation time.

**Supplementary Information:**

The online version contains supplementary material available at 10.1186/s40001-024-01921-5.

## Background

The testis is a highly dynamic, heterogeneity organ and it contains different cell types, seminiferous tubule stages, and cell structure. Cross-sectioned seminiferous tubules contain several germ cell types, which are involved in various cell biological processes ranging from proliferation, meiosis, and differentiation. Although recent advances in molecular biology and biochemistry, such as single-cell sequencing [1, 2], have enabled the elaboration of spermatogenesis, the study of the microenvironment and the spatial localization of cells and molecules still rely on morphology studies. Therefore, testis function study still depends on the observation of the histology section. For these reason, the accurate histological assessment of testes is essential.

Fixatives are the foundation of histopathology based on their ability to preserve tissue in a stable condition, thus enabling the long-term study of cellular structure and tissue composition. Formaldehyde, which forms covalent chemical bonds between protein molecular structures, is the most widely used fixative component [3]. A representative formaldehyde fixative is 10% (*v*/*v*) neutral buffered formalin (NBF), which contains 4% formaldehyde and phosphate-buffered saline (PBS). For most tissue types, 24 h in NBF is the standard for pathologists; however, the fixative and the fixation time in testicular histomorphology are debatable, which may depend on the tissue size and tissue type used in the study [4]. Bouin’s fluid (BF) has been demonstrated to preserved testes morphology better than 10% NBF [5, 6]. In 2002, the Society of Toxicologic Pathology recommended using modified Davidsons Fluid (mDF) to fix testes, because it can preserve high-quality morphological detail, although they recommended BF fixators in the early years [7]. So far, only a few studies have focused on the difference between various fixatives of testis [8–11]; also in addition, the lack of quantitative evidence makes the optimal testis fixation fluid and fixation time still controversial.

High-quality tissue sections are required for the histopathological observation or other downstream experiments, such as RNA scope, spatial transcriptomics [12], NanoString digital spatial profiling (DSP) [13] and protein detection or quantification techniques, such as immunofluorescence and immunohistochemistry (IHC) or other digital pathology methods. The key to preparing a high-quality tissue section is to find an appropriate fixative and fixation time. Different fixatives and fixation time affect the integrity of the tissue structures [4]. The assessment of testicular histomorphology is crucial for studying the male reproductive system.

Due to their phylogenetic relatability and physiological similarity to humans, the mouse (Mus musculus) has long been a model for human biology and disease [14]. Despite the genetic and epigenetic differences between mice and humans [15], C57BL/6 mice are the most used inbred strains in research. Therefore, appropriate histomorphology methods for mouse testes could benefit the study of male reproductive system diseases and biology.

The present study aimed to identify the optimal fixation solution and time for mouse testes through quantitative analysis, and prepare high-quality histopathological sections, thereby laying a suitable foundation for the pathological study of male infertility-related diseases. In this study, three types of fixatives and fixation timepoints were used to treat the testicular tissues on paraffin sections. Hematoxylin and eosin (H&E) staining, glycogen, Periodic acid Schiff-hematoxylin (PAS-h) staining, and IHC staining were used to observe the morphological structure, seminiferous epithelial cycle, and antigen expression status.

## Methods

### Animal and tissue collection

A total of 25 C57BL/6 mice (50 testes), 8–9-week-old, weighing 23–25 g, were provided by the Liaoning Provincial Laboratory Animal Center. Animal experiments were approved by the Laboratory Animal Ethics Committee. After anesthetizing mice with 2% sodium pentobarbital, euthanasia was performed using cervical dislocation, and then, the testes were removed carefully, and samples were handled gently to minimize damage to the soft seminiferous tissue. Experimental design and group information are shown in Supplemental Table 1.

### Tissue allocation, fixation, embedding, and sectioning

To exclude the differences between mice, we employed different fixatives to fix the bilateral testis, i.e., one testis was fixed with 10% NBF/BF, and the contralateral testis was fixed with BF/mDF. Testes from each sample were fixed by immersion in 10 mL of 10% NBF (Sigma, HT5011), Bouin’s Fluid (Sigma, HT10132), and mDF (30% of a 37–40% solution of formaldehyde, 15% ethanol, 5% glacial acetic acid, and 50% distilled H_2_O) for 8, 12, 24, and 48 h at room temperature with gentle rocking. The fixed testes were subjected to gradient alcohol dehydration: 75% ethanol overnight, 85% ethanol for 4 h, 95% ethanol for 1.5 h, anhydrous ethanol for 1 h, xylene clearing for 20 min, and wax immersion for 2 h. Then, wax blocks were prepared and 4-mm sections were cut from the middle portion of each sample. The sections were heated in an oven at 60 ℃ for 40 min, dewaxed in xylene, and rehydrated in decreasing concentrations of ethanol (100%, 90%, 70%, and 50%) before proceeding with coming staining.

### Histological staining

The sections from the fixed samples were stained with H&E (Hematoxylin, Beyotime, C0107; Eosin, Beyotime, C0109) and PAS-h (Periodic Acid–Schiff Staining Kit, Beyotime, C0142S). For H&E staining, sections were incubated in hematoxylin for 2 min, de-stained (1% hydrochloric acid in 70% ethanol) for 5 s, washed in 80% ethanol for 1 min, followed by a brief incubation (1 s) in eosin before dehydration in ethanol (95% and 100%) and clearing in xylene. For PAS-h, the sections were washed in distilled water for 1–2 min, oxidized in periodate for 30 min, washed in distilled water, shaken to remove excess water, incubated in PAS-h staining solution for 30 min, rinsed in running water, stained with hematoxylin, rinsed in running water, dehydrated in gradient alcohol, and sealed in neutral gum.

### IHC using diaminobenzidine

All IHC experiments were conducted two slices for each individual sample. Sections with adequate integrity were selected for IHC, wherein they were fixed in 10% NBF, BF, and mDF for 12 h for the following antibody targets: synaptonemal complexes 3 (Sycp3). After dewaxing and rehydration, sections were subjected to heat-induced antigen retrieval with microwave heating for 5 min in citrate buffer with 0.05% Tween 20 (Citrate Antigen Retrieval Solution, Beyotime, P0081; Tween-20 Beyotime, ST825). To detect Sycp3 proteins, sections were blocked with endogenous peroxidase blocking solution (Endogenous Peroxidase Blocking Buffer, Beyotime, P0100A) at room temperature for 30 min and then in 5% normal goat serum (Goat Serum, Beyotime, C0265) in phosphate-buffered saline (PBS; 135 mM NaCl, 4.7 mM KCl, 10 mM Na_2_HPO_4_, 2 mM NaH_2_PO_4_, pH 7.3). Sections were incubated with primary antibody overnight at 4 ℃ (1:100; SCP3-D-1, Santa Cruz, sc-74569) and then with secondary antibody (Santa Cruz, m-IgGκ BP-HRP, sc-516102) at 37 ℃ for 40 min. After performing the previous steps, sections were washed six times in PBS and treated with 3,3ʹ-diaminobenzidine (DAB) substrate kit (Abcam, ab64238) for IHC staining detection. Hematoxylin was used to re-stain the nucleus, and neutral gum was used to seal the slides.

### Cross-sectional analysis and data processing

After HE, IHC, and PAS-h staining, the sections were scanned at × 40 magnification using a Leica Aperio^®^ ScanScope CS2 scanner, and the results were loaded on the ImageScope (version 12.4.0.5043) for digital analysis. To evaluate the thickness of the spermatogenic epithelium, three distinct measurements were derived from independent stage VI–VIII tubules, extending from the tubule periphery to the region encompassing elongated spermatids. The mean value of these measurements was subsequently utilized as the tubule's thickness; subsequently, 298 individual tubules from 30 sections were measured. For PAS-h staining sections, the region of stage VII was manually annotated from each individual section; 10 sections and 100 stage VII individual tubules were evaluated. The evaluation of the seminiferous epithelial cycle was based on the binary decision key for rapid identification of specific stages of spermatogenesis, as described previously [16]. For IHC staining, the random stage of the seminiferous tubule was annotated for each section, i.e., 160 individual tubules in 16 sections. Next, we used the Leica Aperio Positive Pixel Count v9 algorithm to quantify the positive staining of DAB and glycogen, respectively (Figs. 3 and 5). The algorithm automatically calculated the positive DAB and glycogen staining of the annotated regions into three different pixels type: strong positive (Sp) (red), positive (p) (orange), and weak positive (Wp), (yellow). Hematoxylin was represented by blue-negative cell pixels. The positivity (%) was defined as [Positivity (%) = (Wp + p + Sp)/Ntotal × 100], where Ntotal is the total number of both positive and negative pixels in the annotated regions. All the data generated from the algorithm were imported to R (4.0.6) for statistical analysis and data visualization.

### Statistical analyses

One-way analysis of variance (ANOVA) was used to detect the effect of fixative and duration on seminiferous epithelial thickness and positivity of DAB and PAS-h. Data are shown as mean ± standard deviation (SD), and statistical significance was defined as ( ∗)*P* < 0.05, (∗ ∗)*P* < 0.01, (∗ ∗ ∗)*P* < 0.001, (∗ ∗  ∗ ∗)*P* < 0.0001.

## Results

### Testicular morphology assignment of three fixatives

**Fig. 1:**
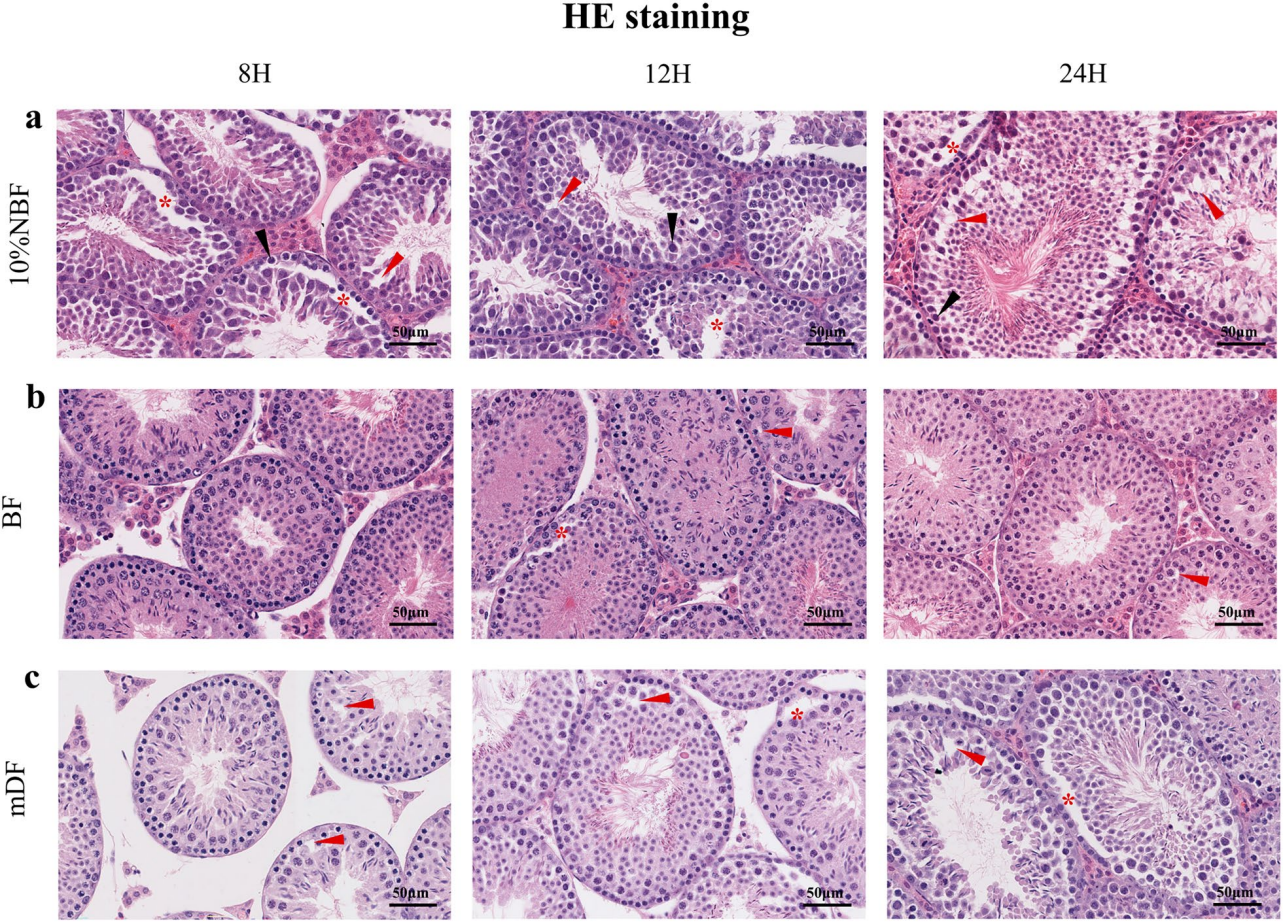
Representative images of hematoxylin and eosin (HE) stained mouse testis sections prepared with different fixatives. Before hematoxylin and eosin (H&E) staining mouse testis samples were fixed in 10%NBF (10% neutral buffered formalin) (**a**), BF (Bouin’s fixative) (**b**) and mDF (modified Davidson’s fluid) (**c**) solution for 8 h, 12 h, and 24 h. Arrows and asterisks highlight area of artefact including: disrupted seminiferous epithelium integrity (blank space; red asterisks), cell nuclei condensation (black arrow), and disrupted cell plasma integrity (blank space; red arrow). Bars = 50 μm

**Fig. 2:**
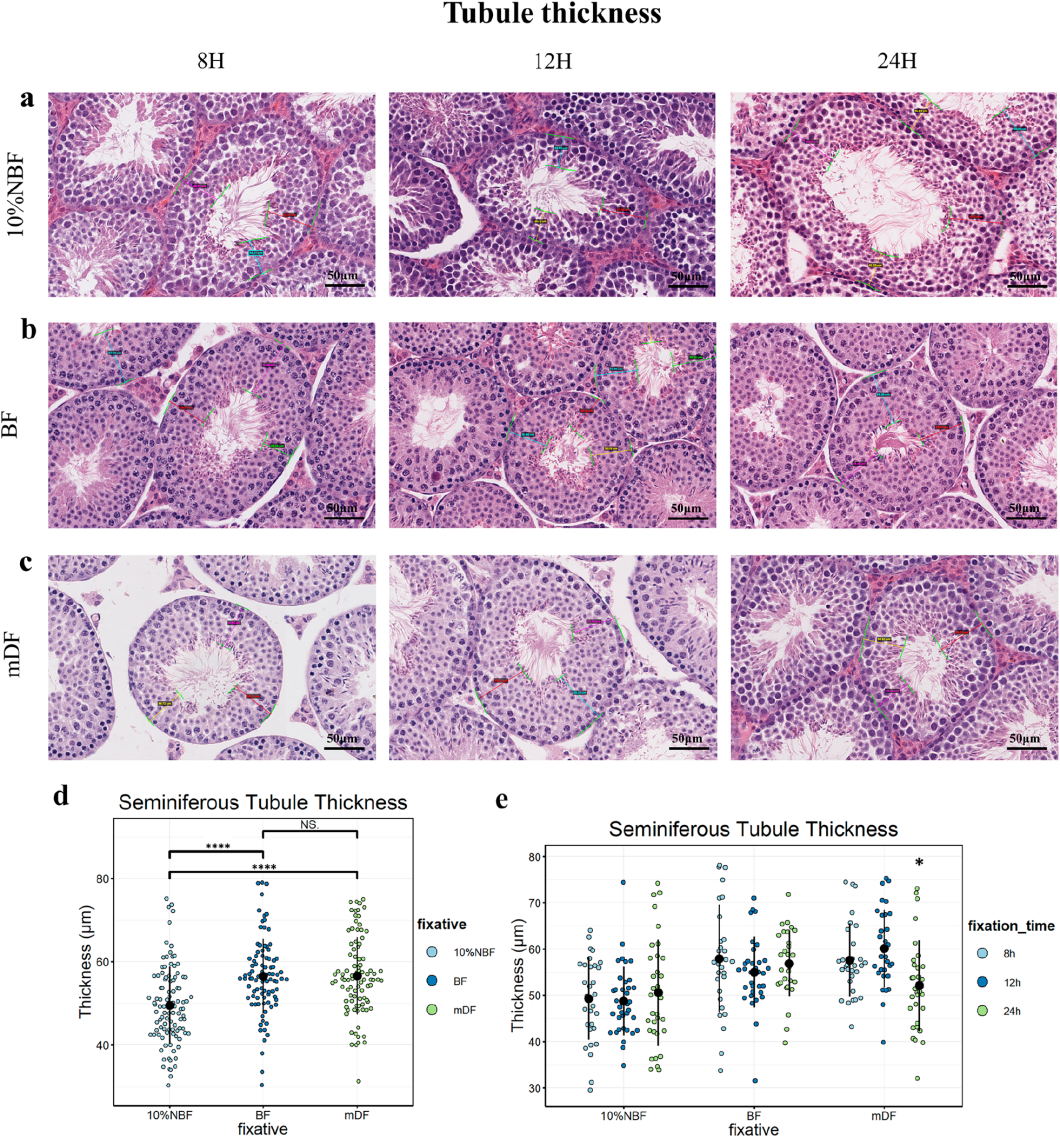
Measurements of seminiferous tubule thickness. **a**–**c** Representative images of measured stage VI–VIII tubules. The average thickness of each tubule was generated from three lengths (from the periphery of the tubule to elongated spermatids), which are annotated by colored lines and numbers. **d** Dot plots show the thickness of the three fixatives, all fixation times were included. **e** Dot plots show thickness between fixatives at different durations. Each dot represents an independent stage VI–VIII tubule, data represent the mean ± standard deviation (SD) of 298 tubules from 30 sections, which were added as a pointrange. One-way ANOVA was used to detect the effect of fixative and duration on seminiferous epithelial thickness, significance was defined as ( ∗)*P* < 0.05, (∗ ∗)*P* < 0.01, (∗ ∗ ∗)*P* < 0.001, (∗ ∗  ∗ ∗)*P* < 0.0001

Fixation of the mouse testis in 10% NBF resulted in distinct chromatin condensation of cell nuclei, thereby causing blurred nuclear structure, due to which the autosome and heterochromatin were indistinguishable. 10% NBF also caused cytoplasmic, seminiferous tubule, and whole testis shrinkage, i.e., deformation of the seminiferous tubule, affecting seminiferous epithelium integrity and creating artifacts (Fig. 1a). In BF and mDF, seminiferous tubule and cell morphological integrity were preserved. BF caused less cytoplasmic shrinkage than mDF (Fig. 1b, c). The thickness of seminiferous epithelium was used to assess seminiferous epithelial shrinkage (Fig. 2) (Supplemental Table 2). While observing the duration of fixation (8, 12, and 24 h) for mDF fixative samples, we found that the fixation time exceeding 24 h resulted in severe shrinkage of the spermatogenic epithelium (Fig. 2e) (Supplemental Table 2). Interestingly, 10% NBF and BF fixatives did not have this outcome. In addition, the analysis of the tubule thickness of the three fixations (including all fixation times) revealed that 10% NBF fixations resulted in significant shrinkage of the spermatogenic epithelium thickness (Fig. 2d). Subsequently, we measured a total of 298 tubules from 30 sections of stage VI to the middle of stage VIII tubules, and found that the thickness of spermatogenic epithelium in 10% NBF was 49.83 ± 9.73 (8 h), 48.75 ± 7.33 (12 h), 50.57 ± 11.32 (24 h); in BF was 60.93 ± 14.83 (8 h), 54.99 ± 7.55 (12 h), 62.37 ± 15.30 (24 h); in mDF was 59.64 ± 10.88 (8 h), 60.80 ± 8.99 (12 h), 53.47 ± 12.34 (24 h). Based on our data, 10% NBF caused shrinkage in 15–17% of spermatogenic epithelium, relatively. We also observed that the artifacts increase with fixation time irrespective of the types of fixatives (Fig. 1a, b, c), despite the low level of artifacts in BF fixed samples. Based on these data, we focused on 12 h fixated samples for further study.

### PAS-h staining and mouse seminiferous tubule staging

**Fig. 3:**
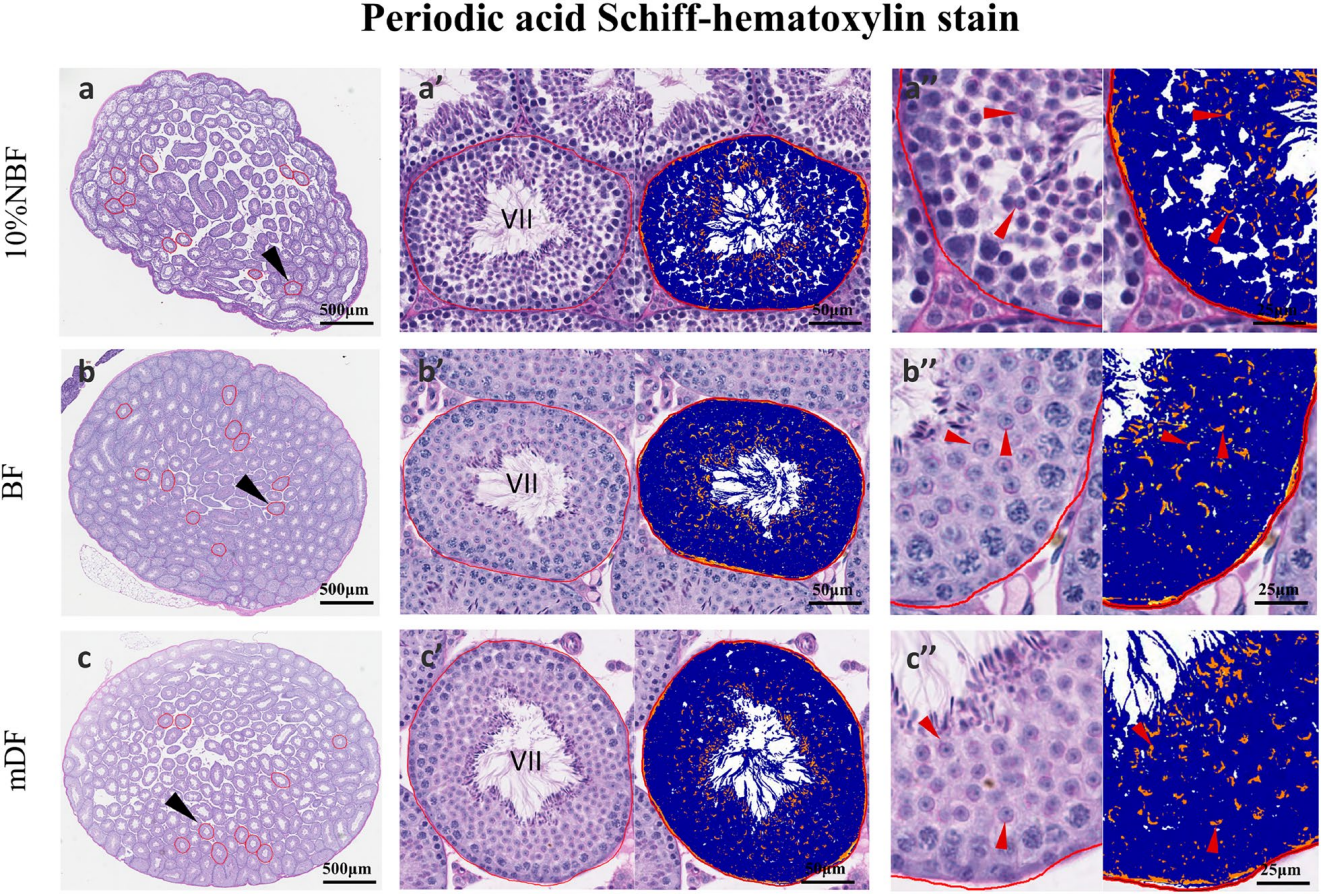
Periodic acid Schiff–hematoxylin (PAS-h) stain of mouse testis tissue prepared with different fixatives. Periodic acid Schiff–hematoxylin (PAS-h) staining was performed on the mouse testis, samples were fixed in 10%NBF (**a**–**a**″), BF (**b**–**b**″), and mDF (**c**–**c**″) for 12 h, the red line annotate each stage VII tubules. **a**, **b**, **c** (left panel): representative testis sections, tubules that require further demonstrated are point out by black arrows, bars = 500 μm; **a**ʹ, **b**ʹ, **c**ʹ (middle panel): a representative stage VII seminiferous tubule, the original (left) and markup (right) images, bars = 50 μm; **a**″, **b**″, **c**″ (right panel): further magnification of the tubule, red arrows point out the arcuate acrosomal caps, bars = 25 μm. The markup image was generated by Leica Aperio Positive Pixel Count v9 algorithm; positivity pixels are represented by: strong positive (Sp) (red), positive (p) (orange), and weak positive (Wp), (yellow)

**Fig. 4:**
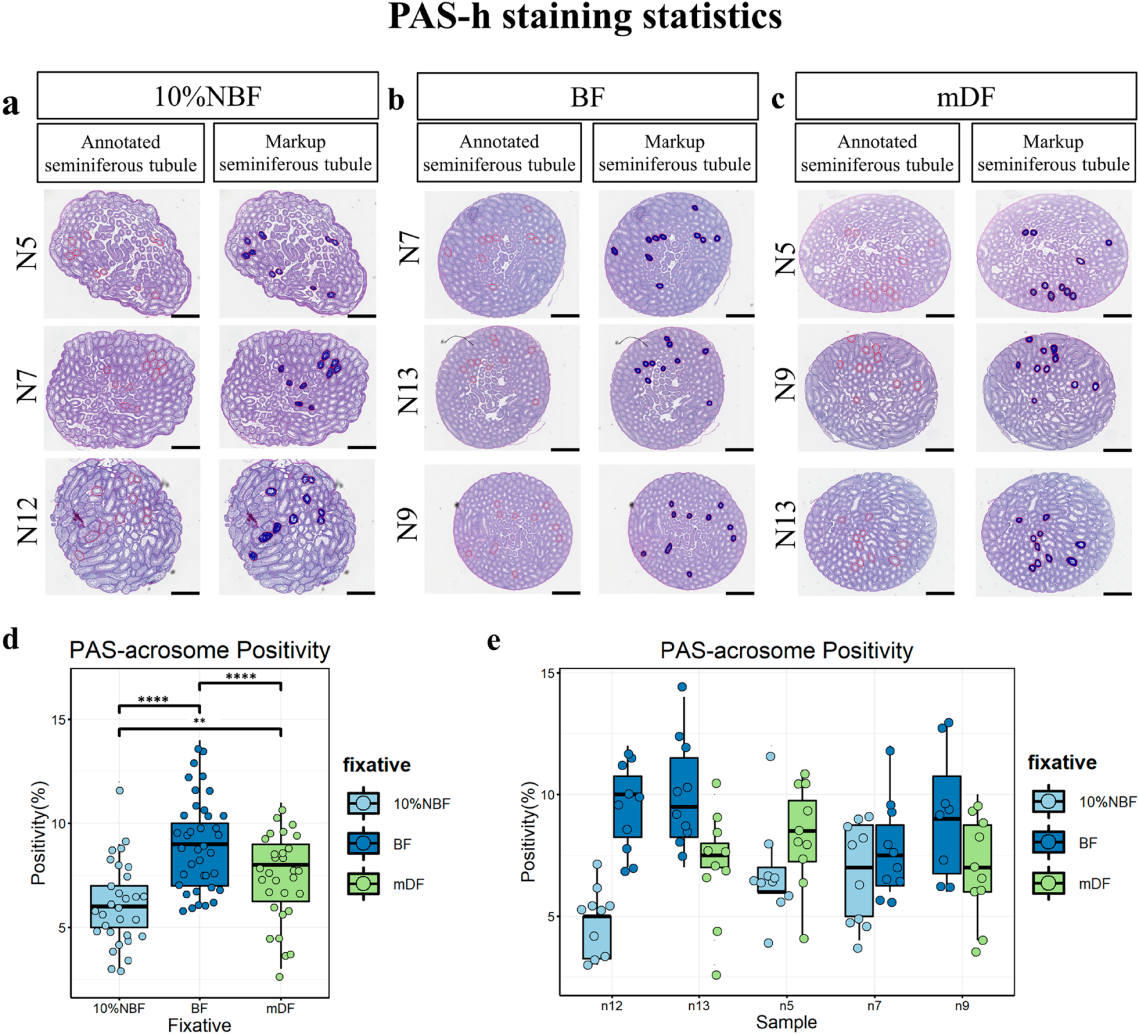
Acrosomic granules assessments of fixative conditions using PAS-h stained mouse testis sections. Representative testis sections from different samples were assessed after performing PAS-h. Samples were fixed in 10%NBF (**a**), BF (**b**), and mDF (**c**) for 12 h, the red line annotate each stage VII tubule that needs to be analyzed, positive data were collected from each markup tubule. D, E Box plot show the Sycp3 positivity (%) of acrosomic granules of different fixatives, arranged by fixatives (**d**) and samples (**e**) (Each individual box represents data collection from one testis of the corresponding sample). Data represent the mean ± SD of 100 seminiferous tubules from 5 mouse. (∗ ∗)*P* < 0.01, (∗ ∗ ∗)*P* < 0.001, (∗ ∗  ∗ ∗)*P* < 0.0001, generated by One-Way ANOVA. Scale bar = 50 μm

To stage seminiferous tubules and assess the difference in spermatid acrosome morphology in three fixatives, 10% NBF, BF, and mDF fixed samples (12 h) were subject to PAS-h. To exclude the differences between different stages of seminiferous tubules, we manually annotated 100 stage VII (10 tubules/testis) seminiferous tubules for analysis. The results showed that the acrosome cap of the samples fixed with 10% NBF fixative was irregular, and the acrosomic granules were clumped, which made it difficult to identify the tubule period (Fig. 3a–a″). Those samples fixed with BF and mDF fixatives showed acrosome and spermatid morphology: arcuate acrosomal caps were clear and plump (Fig. 3bʹ–b″, cʹ–c″). Then, Leica Aperio Positive Pixel Count v9 was employed, and magenta acrosomal caps were well detected and quantified (Fig. 4). The results showed that the positivity (%) of acrosomic granules was significantly different between samples with three fixatives (Fig. 4d, e) (Supplemental Table 3). For a stage VII seminiferous tubule, the positivity (%) of acrosomic granules in 10% NBF, BF, and mDF fixative was 6.06 ± 2.03, 9.55 ± 2.95, and 7.43 ± 2.06, respectively.

### Different fixatives influence IHC staining

**Fig. 5:**
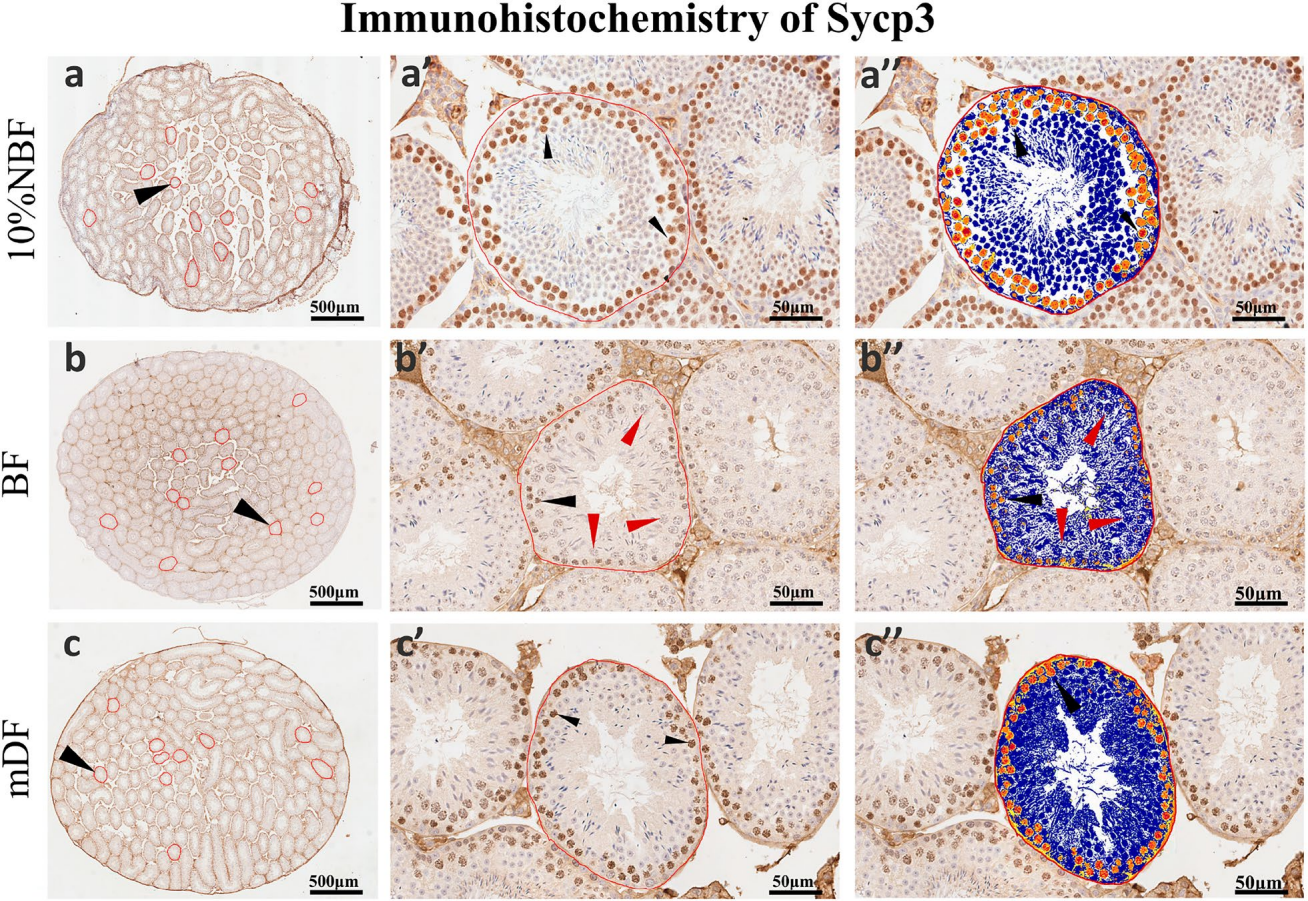
Detection of Sycp3 using immunohistochemistry (IHC) in mouse testis fixed with different fixatives. Following the fixation of testis in 10%NBF (**a**–**a**″), BF (**b**–**b**″) and mDF (**c**–**c**″) for 12 h, immunohistochemistry (IHC) was implemented on mouse testis sections. **a**, **b**, **c** (left panel): Representative testis sections, bars = 500 μm; **a**ʹ–**c**ʹ (middle panel) and **a**″–**c**″ (right panel; markup): Representative seminiferous tubules, black arrows highlighted the Sycp3 (a meiosis marker), which was detected in spermatocytes. Red arrows in B2 and B3 indicate poor DAB coloration in BF-fixed samples. The markup image was generated by Leica Aperio Positive Pixel Count v9 algorithm; positivity pixels are represented by: strong positive (Sp) (red), positive (p) (orange), and weak positive (Wp), (yellow)

**Fig. 6:**
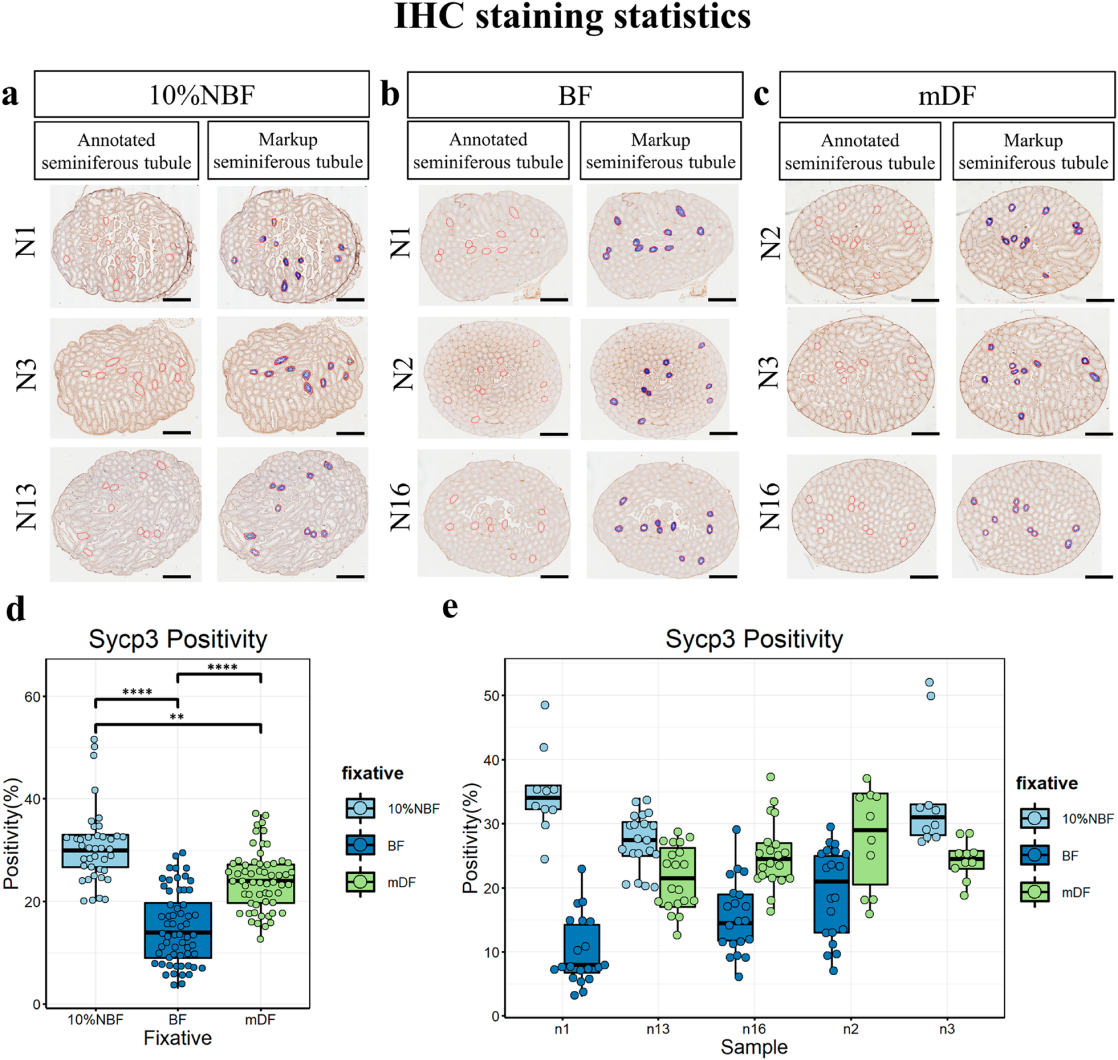
IHC positivity assessments of mouse testis sections were preserved with different fixatives. Representative testis sections from different samples were analyzed after performing IHC. Samples were fixed in 10%NBF (**a**), BF (**b**), and mDF (**c**) for 12 h, the red line annotate random tubule that needs to be analyzed, positive data were collected from each markup tubule. **D**, **E** Box plot show the Sycp3 positivity (%) of different fixatives, arranged by fixatives (**d**) and samples (**e**) (Each individual box represents data collection from one testis of the corresponding sample). Data represent the mean ± SD of 160 seminiferous tubules from 5 mouse. (∗ ∗)*P* < 0.01, (∗ ∗  ∗ ∗)*P* < 0.0001, generated by One-Way ANOVA. Scale bar = 50 μm

To evaluate the immunolabeling efficiency of fixatives, 10%NBF, BF, and mDF fixed samples (12 h) were subject to IHC, using DAB for visualization (Fig. 5). Sycp3 is a meiosis marker widely expressed in spermatogenic epithelium [17–19]. Herein, it was used to evaluate the efficiency of IHC staining. The positivity (%) of Sycp3 was calculated using Leica Aperio Positive suchPixel Count v9. The results showed that the highest IHC staining efficiency was the sections fixed with 10% NBF, and an approximate effect could be achieved with mDF fixative. 10% NBF and mDF showed a good performance in IHC staining, Sycp3 was well localized in the nuclei of spermatocytes, from preleptotene spermatocytes to their meiotic division. (Fig. 5a–a″, c–c″). However, it was poorly detected in spermatocytes fixed with BF, especially in late stage of spermatocytes (Fig. 5b–b″). In summary, BF yielded the worst efficiency for IHC staining (Fig. 6d, e) (Supplemental Table 4). For a seminiferous tubule, the positivity (%) of Sycp3 in 10% NBF, BF, and mDF was 28.07 ± 8.86, 14.93 ± 6.51, and 22.83 ± 5.45, respectively.

## Discussion

The present study aimed to identify the fixatives and fixation time that not only preserved testis tissue morphology but also enabled other downstream histological molecular methods, involving proteins, nucleic acids, and other subcellular organelle detection. Some studies have shown that effective preservation is based on the tissue type, and different fixatives can lead to different morphologies on the same tissue [20, 21]. Hence, in this study, we tested three types of fixatives commonly used in the testis. For different fixation periods, we observed that fixation time of 24 h resulted in morphological damage of the testicle, including but not limited to the shrinkage of seminiferous tubule and testicle volume, seminiferous epithelium damage, and artifact within seminiferous tubules, although this phenomenon was less severe in the BF fixative. Therefore, based on this study, we recommended that while fixing whole mouse testis, the fixation time should be ≤ 12 h and attention should be on the puncture in the long axis in tunica albuginea. On one hand, it can effectively reduce the experimental time; on the other hand, it can reduce the tissue damage caused by the extension of fixation duration. For different fixatives, we observed that fixation with BF can well preserve the morphology of the seminiferous tubules and, in most cases, was capable of reducing the artifacts, but causing testicle volume shrinkage. Conversely, mDF resulted in a small degree of cytoplasmic shrinkage but retained testicular and tubule volume. Typically, the side effects of both fixatives on testicular morphology were mild. The current study showed that 10% NBF caused maximal morphological damage among the three fixatives. Shrinkage introduced by NBF could be a severe problem for the study of the testis. Thus, current evidence suggests that the preservation of testis morphology is crucial for many downstream molecular biology experiments, such as IHC, RNA–FISH (fluorescence in situ hybridization), spatial transcriptomics, and spatial proteomics, spatial metabolomics [12, 13, 22, 23].

Spermatogenesis is a cyclic, continuous process. Within each epithelial area, various stages of germ cells are arranged orderly according to the developmental trajectory. Cross-sectioning can capture a part of this continuous process and define it as a specific stage of the epithelial cycle [24–26]. Hence, the identification of the epithelial stages is essential in the study of male reproductive biology. Due to the specificity of acrosome development, PAS-h staining is an effective tool for marking the stage of acrosomic granules. In this study, we demonstrated that PAS-h staining located the acrosome of the samples fixed by the three fixatives; however, the positivity (%) of acrosomic granules was different. Interestingly, 10% NBF caused cell shrinkage, making it difficult to distinguish the tubule epithelial stages. This was mild in most cases of samples fixed with BF and mdF fixatives, which renders these two fixatives ideal for the identification of the epithelial stages. Sycp3 is a typical marker of spermatocyte meiosis, used to evaluate the preservation of proteins using three fixation fluids. The current results showed that samples fixed with 10% NBF had the highest positivity in IHC staining, followed by mDF. BF has demonstrated the lowest IHC staining efficiency, which was consistent with some previous studies on other tissues [4], this phenomenon was related to the concentration of formaldehyde and picric acid in the fixative [27]. Since Sycp3 is mainly located in the nucleus, and 10% NBF can cause severe cytoplasmic shrinkage, whether it will affect the detection and localization of cytoplasmic proteins needs to be investigated further.

Previous studies have shown that the ideal fixative should enable nucleic acid preservation. NBF interacts with nuclear material and leads to poor detection [28], as well as degradation of the nucleic acid [29]. Nonetheless, comparative studies on nucleic acid preservation of fixatives are yet scarce, but there is a consensus that the preservation of DNA in paraffin-embedded samples is superior to RNA preservation, because high temperatures are required during the preparation of paraffin sections that disrupt the RNA structure [30, 31]. We also found that 10% NBF caused more tissue hardening than the other two fixatives; this feature increases with the prolonged fixation time, causing sectioning difficulty. Another study suggested that acetic acid alleviates the hardening of tissues caused by alcohol [32]. In fact, although the histomorphology was an old question, novel biological techniques have made it necessary to revisit it and improve them accordingly.

## Conclusions

We evaluated the performance of three commonly used fixatives, 10% NBF, mDF, and BF in HE, PAS-h, and IHC staining, which were used for tissue morphology assessment, seminiferous tubule staging and protein detection. We also discussed the effect of fixation time on testicular morphology. Critically, we used a new way of quantitative analysis to evaluate the efficacy of different fixations for acrosome detection. However, our study was limited and did not discuss the preservation of biomacromolecules other than proteins, previous studies have not shown the difference between different preservation protocols in the preservation of DNA or RNA in testis, which needs to be further confirmed by more complex experimental designs and studies. Based on our current data, we suggested that for mouse testes, fixation time should be for ≤ 12 h after the puncture in tunica albuginea, to reduce the tissue damage caused by long duration of fixation and reduce the experimental time. BF can be used when the researcher needs to grade the seminiferous tubule, and mDF should be used when other proteinic downstream experiments are required; 10% NBF was not recommended for testicular morphological studies.

### Supplementary Information

Supplementary Material 1: Table 1: experimental design.

Supplementary Material 2: Table 2: thickness of seminiferous epithelium raw data.

Supplementary Material 3: Table 3: Positivity (%) of acrosomic granules raw data.

Supplementary Material 4: Table 4: Positivity (%) of Sycp3 raw data.

## Data Availability

All data generated or analyzed during this study are included in this article.
